# Neuropathological and behavioral characterization of aged *Grn* R493X progranulin-deficient frontotemporal dementia knockin mice

**DOI:** 10.1186/s40478-021-01158-x

**Published:** 2021-04-01

**Authors:** Jonathan Frew, Haakon Berge Nygaard

**Affiliations:** Djavad Mowafaghian Centre for Brain Health and Division of Neurology, University of British Columbia, 2215 Wesbrook Mall, Vancouver, BC Canada

**Keywords:** Frontotemporal dementia, Progranulin, R493X, Mouse model, Lysosomal dysfunction, Open field, Microgliosis, Astrogliosis, TDP-43, Neurodegeneration

## Abstract

Frontotemporal lobar degeneration (FTLD) causes a spectrum of clinical presentations of frontotemporal dementia (FTD), including progressive changes in behavior, personality, executive function, and language. Up to 20% of familial FTLD cases are caused by progranulin (*GRN*) haploinsufficiency (FTD-*GRN*), with one of the most common causal variant being a nonsense mutation at arginine 493 (R493X). Recently, a genetic knockin FTD-*GRN* mouse model was generated bearing this *Grn*^*R493X*^ mutation, at the analogous arginine in murine *Grn*. Aged, homozygous *Grn*^*R493X*^ mice (*Grn*^*R493X/R493X*^) have been shown to phenotypically replicate several neuropathological hallmarks previously demonstrated in *Grn* null mice. We conducted a comprehensive neuropathological and behavioral assessment of 18 month old *Grn*^*R493X/R493X*^ mice, observing a striking lysosomal dysfunction and thalamic neurodegeneration not previously described in this model, as well as a male-specific increase in generalized anxiety. These findings provide additional phenotypic markers of pathogenesis in aged *Grn*^*R493X/R493X*^ mice that will contribute to better defining mechanisms underlying FTD-*GRN*, and offer relevant outcome measures for preclinical efficacy testing of novel therapeutics that target nonsense mutations leading to this devastating disease.

**Supplementary Information:**

The online version contains supplementary material available at 10.1186/s40478-021-01158-x.

## Introduction

The neuropathology observed in patients bearing progranulin (*GRN*) loss-of-function (LOF) mutations is dictated by a gene dosage-dependent effect, with most haploinsufficient individuals developing an early-onset form of frontotemporal dementia (FTD-*GRN*) [4, 14]. Individuals null for *GRN* typically suffer from a rare form of neuronal ceroid lipofuscinosis (NCL), CLN11, with disease onset ranging from teenage years to midlife [22, 31]. The discovery that *GRN*-null individuals develop a lysosomal storage disease has encouraged investigations into the role of progranulin (PGRN) and granulin peptides in regulating lysosomal function [6, 11, 12, 16, 17, 20, 21, 33, 35, 37]. The majority of known neurobiological functions of PGRN have been uncovered through the use of mouse models null for *Grn* (*Grn*^*−/−*^), partially because preclinical models of *Grn* haploinsufficiency do not replicate many of the neuropathological hallmarks observed in either FTD-*GRN* or CLN11. Microglial lysosomal dysfunction and neuronal lipofuscin accumulation are the earliest pathological phenotypes observed in the *Grn*^*−/−*^ mouse brain [16, 24], preceding well-established phenotypes including microgliosis, synaptic loss, and TDP-43 pathology [15, 26, 27, 34, 40, 42]. Recently, the *Grn*^*R493X*^ mouse model was generated to more accurately model FTD-*GRN* by introducing one of the most common human nonsense mutation leading to FTD (R493X) at the analogous mouse *Grn* codon (R504X) [29]. Previous characterization of this nonsense mutant *Grn* model identified several disease phenotypes seen in other *Grn*^*−/−*^ models, but lysosomal dysfunction beyond increases in lipofuscin or degeneration of selective neuronal populations have not yet been identified [29]. We sought to comprehensively characterize behavioral and neuropathological phenotypes in aged *Grn*^*R493X/R493X*^ mice, which is critical to fully using this model for drug discovery and efficacy testing.

## Materials and methods

### Mice and facilities

Animal procedures were approved by the University of British Columbia (UBC) Animal Care and Biosafety Committees. *Grn*^*R493X/R493X*^ mice on a C57BL/6 J background, and C57BL/6 J wildtype mice (*Grn*^+*/*+^) were obtained from Jackson Laboratories (Stock #029919 and #000664, respectively). Mice were housed at the UBC Centre for Disease Modeling barrier facility, with a 12 h light/12 h dark cycle and allowed food and water ad libitum. 

### Genotyping

Mouse ear notch DNA was isolated in Chelex® 100. Briefly, 100 µL Chelex® 100 was added to ear notch, vortexed for 10 s (sec), and pulse spun to ensure tissue was submerged in the solution. Samples were then incubated at 95 °C for 20 min. Following the incubation samples were further agitated by running the bottom of the tubes across metal tube storage racks and centrifuged at 21,000 × *g* for 1.5 min. The polymerase chain reaction (PCR) was run using primers and settings outlined by Jackson Laboratories for stock #029919. The resulting PCR products were resolved on a 2% agarose gel and detected using SafeView Classic (Applied Biological Materials).

### Antibodies

The antibodies used in this study were anti-C1q (Abcam, ab182451, 1:500), anti-DppII (R&D Systems, AF3436, 1:500), anti-Foxp2 (Abcam, ab16046, 1:500), anti-Gfap (STEM CELL Technologies, 60128, 1:500), anti-Iba1 (WAKO, 019-19741, 1:500), anti-Lamp1 (BD Biosciences, 553792, 1:500), anti-Pgrn (R&D Systems, AF2420, 1:1000), TDP-43 (Proteintech, 10782-2-AP, 1:500), anti-Tuj1 (Neuromics, CH23005, 1:500), anti-Vgat (Synaptic Systems, 131011, 1:300), donkey anti-goat IgG (H + L) Alexa Fluor® 488 (Thermo Fisher Scientific, A-11006), donkey anti-rabbit IgG (H + L) Alexa Fluor® 647 (Thermo Fisher Scientific, A-31573), goat anti-rat IgG (H + L) Alexa Fluor® 647 (Thermo Fisher Scientific, A-12247), donkey anti-mouse IgG (H + L) Alexa Fluor® 488 (Thermo Fisher Scientific, A-21202), goat anti-mouse IgY (H + L) Alexa Fluor® 488 (Thermo Fisher Scientific, A-21449) (for immunofluorescence); Ctsd (R&D Systems, AF1029, 1:500), Lamp1 (BD Biosciences, 553792, 1:500), LC3-I/II (Cell Signaling, 2775, 1:1000), Pgrn (R&D Systems, AF2557, 1:100), TDP-43 (Proteintech, 10782-2-AP, 1:1000), p-TDP-43 (Cosmo Bio USA, CAC-TIP-PTD-P03, 1:500), Actin (Novus Biologicals, NB600-532**,** 1:10,000), donkey anti-goat IgG-HRP (R&D Systems, HAF109), donkey anti-sheep IgG-HRP (R&D Systems, HAF016), donkey anti-rat IgG-HRP (R&D Systems, HAF005), goat anti-rabbit IgG-HRP (for western blot).

### Perfusion and tissue processing

Eighteen month old mice were anesthetized using isoflurane and transcardially perfused with phosphate-buffered saline (PBS) solution. Brains were hemisected down the midsagittal plane, with one half immediately flash frozen on dry ice and the other fixed overnight in 4% paraformaldehyde. Frozen halves were stored at − 80 °C until lysate preparation and fixed halves were transferred to PBS + 0.05% azide for long term storage. Fixed hemi-brains were sectioned sagittally (40 µm) using a vibratome (Leica). Sections were also stored in PBS with 0.025% sodium azide at 4 °C until used for immunofluorescence staining. Flash frozen hemi-brains were later thawed, homogenized in 250 µL radioimmunoprecipitation assay (RIPA) lysis buffer (150 mM NaCl, 1 mM EDTA, 1 mM sodium orthovanadate, 1 mM NaF, 1 mM β-glycerophosphate, 2.5 mM sodium pyrophosphate, 1 mM PMSF, 1% NP-40, PhosSTOP, cOmplete mini protease inhibitor), sonicated for 10 s at 20% amplitude and ultracentrifuged at 100,000 × *g* for 20 min at 4 °C. The supernatants were collected as RIPA-soluble extract, and the insoluble protein pellet was further extracted with urea buffer (30 mM Tris–HCl pH 8, 7 M urea, 2 M thiourea, 4% CHAPS, PhosSTOP, cOmplete mini protease inhibitor) and centrifuged at 150,000 × *g* for 45 min at 4 °C. The protein concentration of RIPA-soluble fractions was measured by Bradford, diluted to 1 mg/mL for enzyme-linked immunofluorescent assay (ELISA) and western blots. The insoluble urea fractions were diluted according to the protein concentration of corresponding RIPA fractions.

### Immunofluorescence microscopy

Free-floating sagittal brain sections were blocked and permeabilized with 10% donkey or goat serum (Sigma-Aldrich) in Dulbecco’s PBS (D-PBS) containing 0.1% Triton X-100 (Abcam) for 2 h at room temperature (RT). Primary antibodies were then diluted (see antibody section) in 10% donkey or goat serum in D-PBS and applied to sections overnight at 4 °C with gentle agitation. All Alexa Fluor®-tagged secondary antibodies were used at a dilution of 1:500 at RT for 2 h. The sections were then mounted in 4′,6′-diamidino-2-phenylindole (DAPI) mounting medium (Vector Laboratories). Z-stacks and tilescans were captured with ZEN 2 software using a Zeiss 880 scanning laser confocal microscope using a 40X objective lens. High magnification (100X, 2.5X digital zoom with 40X objective) Tuj1-TDP-43-DAPI co-stained images were captured as single Z-plane images. All other images were obtained through single field 30 µm thick (1 image / 5 µm step) z-stack and z-stack tilescan acquisitions and were processed into maximum intensity projections using ZEN 2 software prior to image quantification performed using the Fiji processing package for ImageJ (National Institutes of Health).

### Western blot

RIPA- and urea-soluble lysates were diluted in 4X sodium dodecyl sulphate–polyacrylamide gel electropheresis (SDS-PAGE) loading buffer + 100 mM DTT and boiled at 95 °C for 5 min. 15 µg of protein from each boiled SDS lysate was separated on 4–15% gradient precast polyacrylamide gel (Bio-Rad), electroblotted onto a nitrocellulose membrane and blocked for 1 h in 5% (w/v) non-fat milk (blocking buffer). Samples from *Grn*^*R493X/R493X*^ and wildtype control mice (*Grn*^+*/*+^) were run on the same precast gel to allow for direct comparison. Membranes were incubated with primary antibodies diluted in blocking buffer overnight at 4 °C, washed three times with tris-buffered saline (TBS) + 0.1% (v/v) Tween-20 (TBS-T), incubated with a 1:10,000 dilution of HRP-conjugated secondary antibody, washed again 3X with TBS-T and incubated with enhanced chemiluminescence substrate (Millipore). Films were developed, scanned, and analyzed using Fiji for densitometry analysis. To reprobe blots with the same species of primary antibody, membranes were stripped with 0.1 N NaOH for 5 min, washed twice with deionized H2O, and incubated for 30 min with non-fat milk blocking buffer before incubation with an additional primary antibody.

### Progranulin ELISA

Pgrn levels in RIPA soluble hemi-brain lysates were determined by ELISA (Adipogen, mouse) using the manufacturer’s protocol. RIPA-soluble lysates (10 mg/mL) were diluted (*Grn*^+*/*+^ 1.5:10 and *Grn*^*R493X/R493X*^ 1:5) in ELISA buffer.

### Behavioral analysis

The open field test was performed to evaluate anxiety in *Grn*^*R493X/R493X*^ and wildtype control mice (*Grn*^+*/*+^). The test consisted of one 10 min trial in a white opaque 40 cm × 40 cm × 30 cm arena (Maze Engineers). The center zone was defined as a square covering 16% of the total area (16 cm × 16 cm central square). The mice were moved to the experimental room at least 1 h before starting the tests. To begin each test, a mouse was introduced to the center of the square and its behavior was captured on video over the course of 10 min. All males were tested prior to any females, and the area was cleaned with 50% ethanol and allowed to dry completely between each test. The duration that each mouse stayed in either the peripheral or central regions was quantified using JWatcher software (UCLA).

### Statistical analysis

All values are expressed as the mean ± SEM. In experiments where two groups were compared, a standard unpaired two-tailed Student’s *t*-test was performed to measure significance. For comparisons of more than two groups, one-way analysis of variance (ANOVA) was used followed by Tukey’s post hoc test. *p *values less than 0.05 were considered significant. Statistical analysis was performed using GraphPad Prism Software, Version 5.0.

## Results

### Brain Pgrn expression in aged ***Grn***^***R493X/R493X***^ mice

**Fig. 1:**
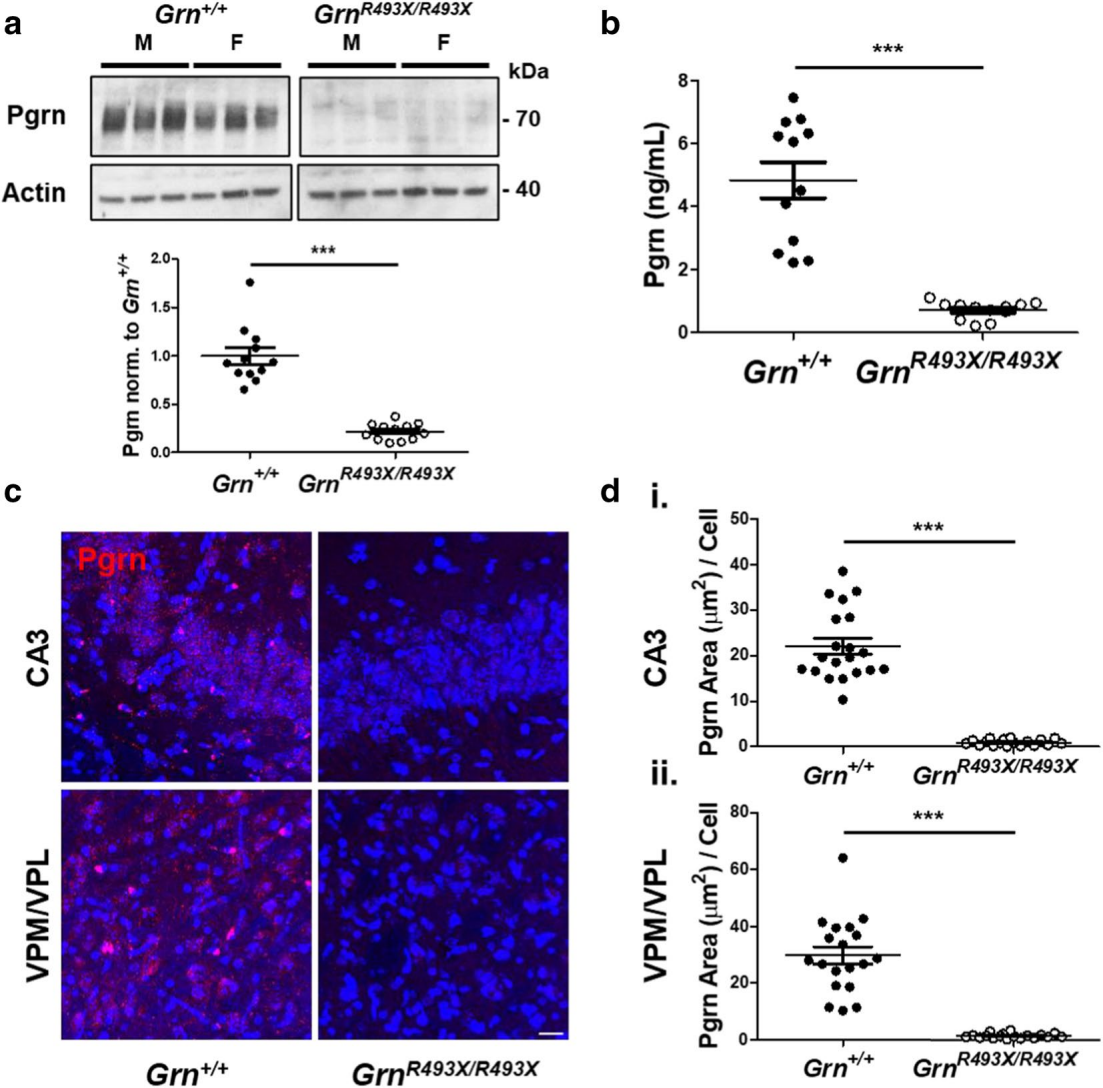
Pgrn expression in the CNS of aged *Grn*^*R493X/R493X*^ mice. 18 month old *Grn*^+*/*+^ and *Grn*^*R493X/R493X*^ hemibrain RIPA-soluble lysate samples were subjected to both Pgrn western blot (**a**) and Pgrn ELISA (**b**). Densitometric quantification of Pgrn western blot signal was normalized to actin loading control. **c**, Pgrn expression was also detected in the hippocampal CA3 and thalamic VPM/VPL regions by immunofluorescence staining of 18 month old *Grn*^+*/*+^ and *Grn*^*R493X/R493X*^ brain sections (scale bar, 20 µm). **d**, Total Pgrn  positive area in CA3 (i) and VPM/VPL (ii) was quantified and normalized to the total number of cells (DAPI, blue). For western blot/ELISA *n* = 6 mice were used per sex/genotype; for immunofluorescence staining *n* = 10 mice were used per sex/genotype (except male *Grn*^*R493X/R493X*^ *n* = 8); values are shown as mean ± SEM; ***p < 0.0001, Student’s *t*-test

We obtained the previously generated FTD-*GRN*/CLN11 mouse model bearing homozygous *Grn* R504X mutations analogous to the human *GRN* R493X mutation for further neuropathological and behavioral characterization [29]. Homozygous introduction of the *Grn*^*R493X*^ premature termination codon (PTC) in these mice was confirmed by PCR (Additional file 1: Fig. S1). Central nervous system (CNS) Pgrn expression in 18 month old *Grn*^+*/*+^ and *Grn*^*R493X/R493X*^ mice was assayed using multiple immunological detection methods, including western blot (Fig. 1a), ELISA (Fig. 1b), and immunofluorescence microscopy (Fig. 1c, d). These results demonstrate that nonsense mutant Pgrn expression is significantly reduced, detecting *Grn*^*R493X/R493X*^ global Pgrn CNS expression levels of 14.7% ± 1.7% (ELISA) and 21.7% ± 2.5% (western blot) relative to *Grn*^+*/*+^ expression levels (Fig. 1a, b). Immunofluorescent quantification of Pgrn expression in hippocampal CA3 and thalamic ventral posteromedial (VPM)/ventral posterolateral(VPL) *Grn*^*R493X/R493X*^ pathological hotspots identified even lower levels of Pgrn, with total Pgrn area per cell of 4.3% ± 0.7% (CA3) and 4.6% ± 0.7% (VPM/VPL) relative to *Grn*^+*/*+^ tissue levels (Fig. 1c, d).

### Lysosomal dysfunction in aged *Grn*^*R493X/R493X*^ mice

**Fig. 2:**
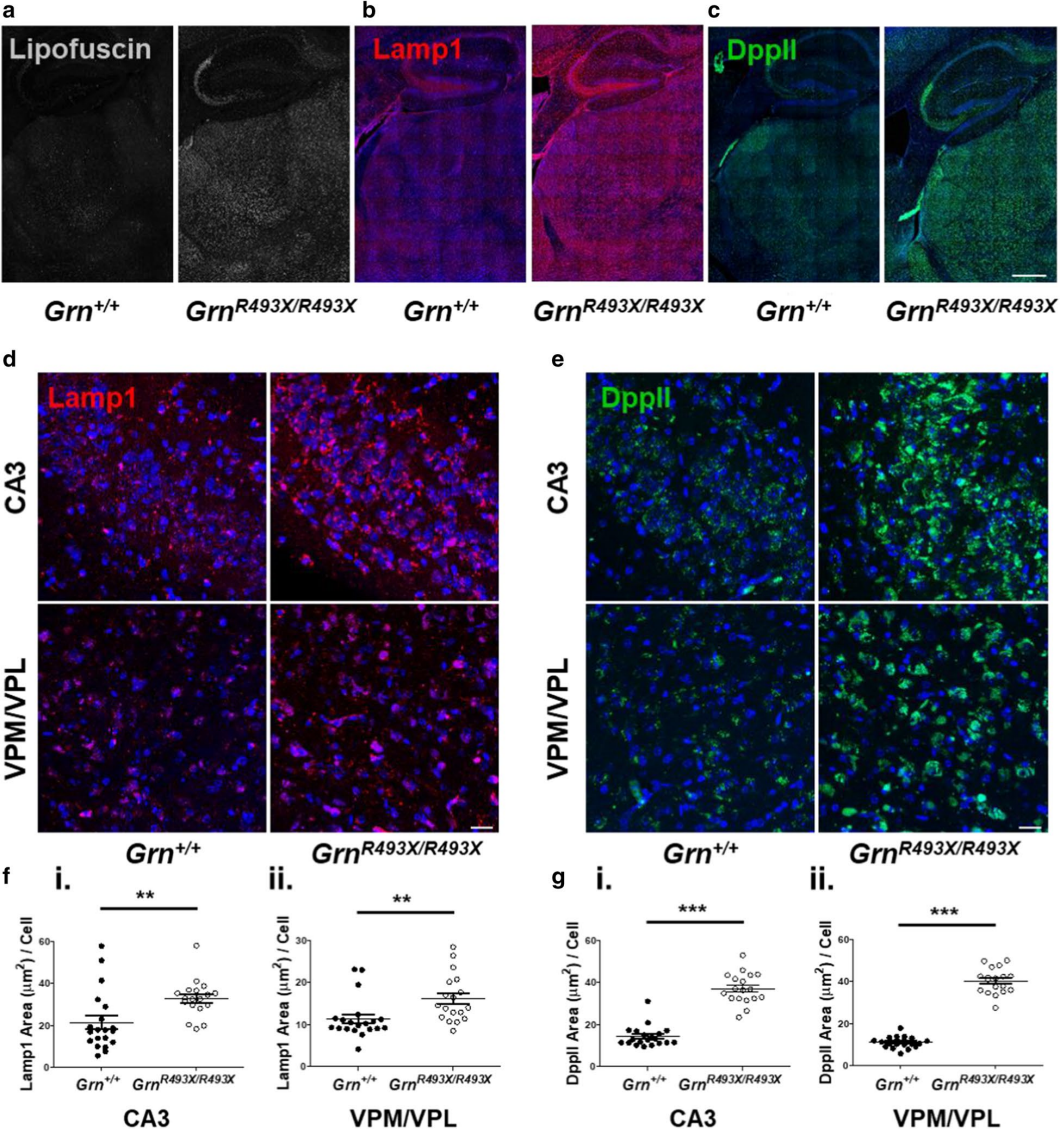
Lysosomal dysfunction in the ventral thalamus and CA3 hippocampal region of aged *Grn*^*R493X/R493X*^ mice. Representative hippocampal/thalamic tilescans highlighting autofluorescent lipofuscin (**a**, green channel), Lamp1 (**b**), and DppII (**c**) accumulation in 18 month old *Grn*^*R493X/R493X*^ mice (scale bar, 500 µm). **d**, **e** Representative Lamp1 and DppII immunofluorescence images of hippocampal CA3 and thalamic VPM/VPL regions in 18 month old *Grn*^+*/*+^ and *Grn*^*R493X/R493X*^ brain sections (scale bar, 20 µm). **f**, **g** Total Lamp1-positive and DppII-positive area in CA3 (i) and VPM/VPL (ii) images were quantified and normalized to the total number of cells (DAPI, blue). For immunofluorescence staining *n* = 10 mice were used per sex/genotype (except male *Grn*^*R493X/R493X*^ *n* = 8); values are shown as mean ± SEM; **p < 0.01, ***p < 0.0001, Student’s *t*-test

Previous studies in *Grn*^*−/−*^ mice have found that CNS lipofuscin accumulation occurs as early as 2–3 months of age [24]. We replicated the thalamic/hippocampal autofluorescent lipofuscin phenotype reported in the *Grn*^*R493X/R493X*^ mouse model [29], observing extensive lipofuscin in 18 month old mice in both the CA3 hippocampal region and thalamus (Fig. 2a). Further, aged R493X knockin mice exhibit increased expression of the lysosomal proteins Lamp1 and DppII, which were also shown to accumulate in these brain regions (Fig. 2b, c). We quantified Lamp1-positive and DppII-positive areas in the thalamus and hippocampus of aged *Grn*^*R493X/R493X*^ mice and observed a significant increase in the expression of these lysosomal markers in the CA3 and VPM/VPL brain regions (Fig. 2d–g). Increased lysosomal vesicle size has been previously reported in several models of *Grn*-deficiency, including *Grn*^*−/−*^ mouse hippocampal neurons [12] and *Grn*^*R493X/*+^ primary cortical neurons [11]. In line with these data, we found that *Grn*^*R493X/R493X*^ CA3 and VPM/VPL regions accumulate significantly larger Lamp1-positive lysosomal vesicles than *Grn*^+*/*+^ control mice (Additional file 1: Fig. S2A, B).

**Fig. 3:**
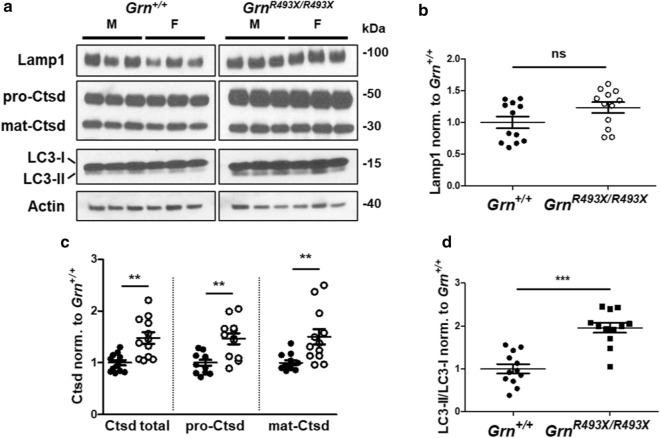
Global dysregulation of lysosomal function in aged *Grn*^*R493X/R493X*^ brain. Representative western blots of hemibrain RIPA-soluble lysates from 18 month old *Grn*^+*/*+^ and *Grn*^*R493X/R493X*^ mice probed with the indicated antibodies (**a**). Expression of Lamp1, total/pro/mat-Ctsd, and LC3I/II in *Grn*^+*/*+^ and *Grn*^*R493X/R493X*^ hemibrain lysates was analyzed by western blotting, using actin as the loading control. Densitometric quantification of brain-wide Lamp1 (**b**) and Ctsd (**c**) expression was normalized to actin and *Grn*^+*/*+^ levels. The LC3 II:LC3 I densitometric expression ratio (**d**) was normalized to *Grn*^+*/*+^ levels. *n* = 6 mice were used per sex/genotype; values are shown as mean ± SEM; ns = not significant, **p < 0.01, ***p < 0.0001, Student’s *t*-test

Aged *Grn*^*−/−*^ mice develop global CNS lysosomal dysfunction, including disrupted autophagosome clearance [6], overexpression of lysosomal enzymes [16], and cytoplasmic accumulation of insoluble TAR DNA-binding protein 43 (TDP-43) [17]. To evaluate CNS-wide lysosomal dysfunction in this model, we conducted a series of western blot assays on RIPA-soluble and -insoluble hemi-brain lysates. Unlike previous findings in *Grn*^*−/−*^ mice [3], Lamp1 protein levels were not found to be significantly increased in the *Grn*^*R493X/R493X*^ brain, although the data trended toward elevated Lamp1 expression (Fig. 3a, b). Increased expression and enzymatic maturation of the lysosomal protease Cathepsin D (Ctsd) has been reproducibly observed in whole *Grn*^*−/−*^ brain tissue [16]. Expression of both the pro- and mature forms of Ctsd were significantly increased in *Grn*^*R493X/R493X*^ brain compared to *Grn*^+*/*+^ (Fig. 3a, c). Lysosomal dysfunction can lead to functional impairments in autophagy, an essential process in maintaining cellular health. Chang et al. identified impaired autophagy pathway signaling in whole cortical tissue obtained from 18 month old *Grn*^*−/−*^ mice, including an approximate 35% increase in the LC3 II:LC3 I ratio, which indicates autophagosome accumulation [6]. Our similarly aged *Grn*^*R493X/R493X*^ mice exhibited a nearly twofold increase in LC3 II:LC3 I over *Grn*^+*/*+^ (Fig. 3a, d), suggesting a dysregulated autophagy phenotype that persists despite the low levels of lysosomal accumulation of partially functional Pgrn-R493X that may occur in these mice [29].

**Fig. 4:**
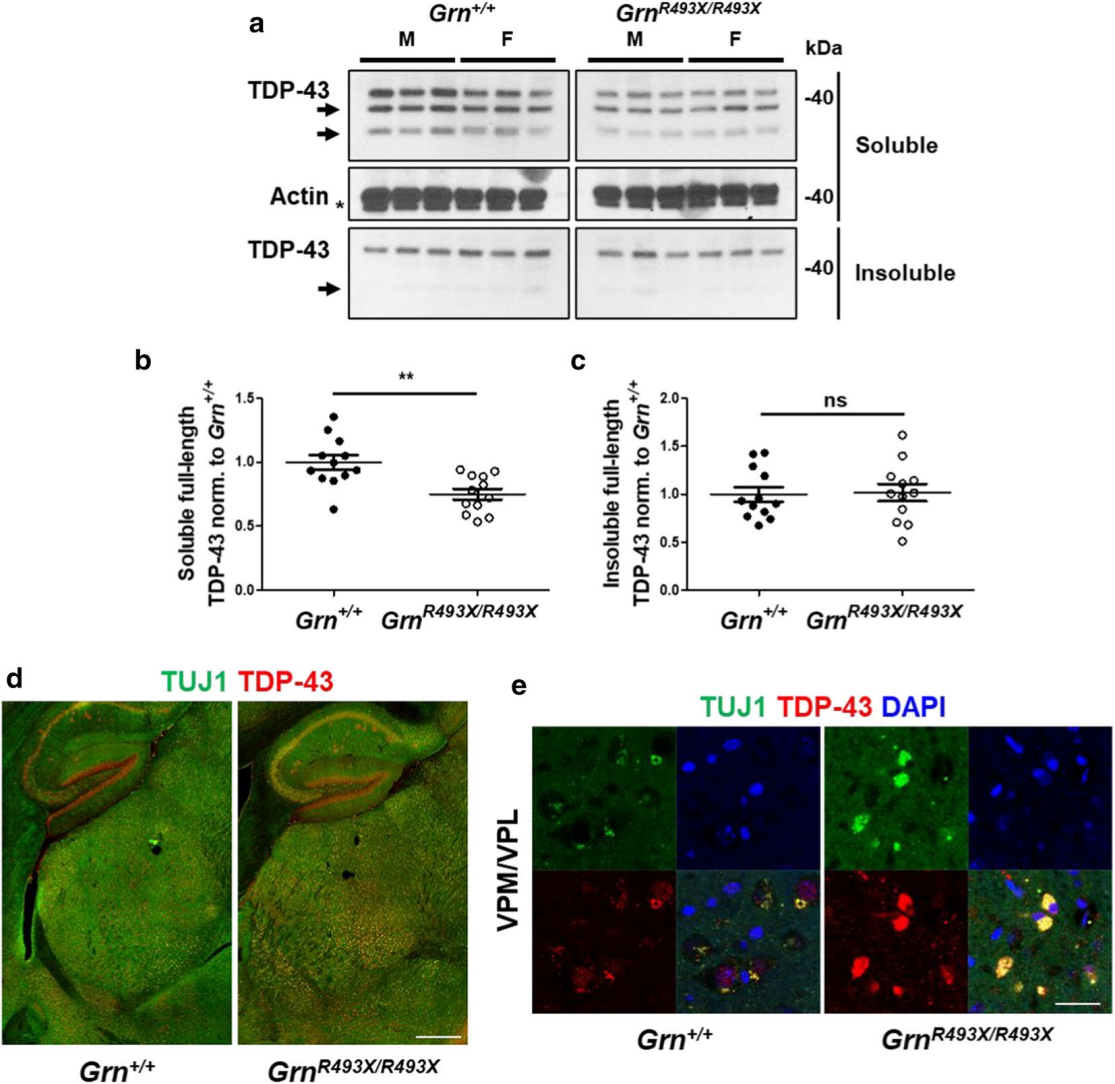
Neuronal TDP-43 proteinopathy is localized to the ventral thalamus of aged *Grn*^*R493X/R493X*^ mice. Representative western blots of hemibrain RIPA-soluble and -insoluble lysates from 18 month old *Grn*^+*/*+^ and *Grn*^*R493X/R493X*^ mice probed for TDP-43 expression. **a** Expression of full-length TDP-43 in *Grn*^+*/*+^ and *Grn*^*R493X/R493X*^ hemibrains in soluble and insoluble lysates was analyzed by western blotting, using RIPA-soluble actin as the loading control (no actin detected in insoluble urea fraction). Arrows indicate TDP-43 CTFs, and the * demarks a remnant TDP-43 signal observed upon reprobing stripped RIPA-soluble TDP-43 blot with actin antibody. Densitometric quantification of brain-wide full-length TDP-43 expression in soluble (**b**) and insoluble (**c**) lysate fractions were normalized to RIPA-soluble actin and *Grn*^+*/*+^ levels. **d** Representative hippocampal/thalamic tilescans highlighting the neuronal (TUJ1, green) TDP-43 (red) proteinopathy phenotype in the ventral thalamus of 18 month old *Grn*^*R493X/R493X*^ mice (scale bar, 500 µm). **e** High magnification images from the thalamic VPM/VPL regions demonstrating neuronal cytoplasmic accumulation of TDP-43 in 18 month old *Grn*^*R493X/R493X*^ mice (DAPI, blue; scale bar, 10 µm). For western analysis *n* = 6 mice were used per sex/genotype; values are shown as mean ± SEM; ns = not significant, **p < 0.01, Student’s *t*-test

Impaired autophagy has been linked to increases in pathological forms of TDP-43, a pathologic hallmark of FTD-*GRN*. Although *Grn*^*−/−*^ mice develop a limited form cytoplasmic/nuclear TDP-43 aggregation slightly resembling histopathology observed in FTD-*GRN* patients [39], biochemical observations in *Grn*^*−/−*^ mice have identified increased full-length and phosphorylated TDP-43 (p-TDP-43) expression in whole-brain RIPA-insoluble fractions [17, 39]. Full-length TDP-43 can be cleaved into aggregation-prone c-terminal fragments (CTFs) that form the major protein component of TDP-43 positive inclusions [1, 10]. 12 month old *Grn*^*R493X/R493X*^ brains were found to contain diffuse cytoplasmic TDP-43/pTDP-43 positivity similar to *Grn*^*−/−*^ mice in select thalamic neurons that was absent in *Grn*^+*/*+^ mice [29]. We reasoned that older mice may display a more robust TDP-43 phenotype. Using a polyclonal N-terminal TDP-43 antibody known to detect multiple forms of TDP-43, including full-length and several truncated CTFs [36], we probed RIPA-soluble and -insoluble hemi-brain lysates for TDP-43 expression. Full-length TDP-43 expression in the soluble fraction was significantly decreased in *Grn*^*R493X/R493X*^ mice (Fig. 4a, b), with a similar trend observed for CTFs (Fig. 4a, arrows). Surprisingly, decreased soluble TDP-43 expression did not correspond with an increase in insoluble TDP-43 levels (Fig. 4a, c). We further probed these lysate fractions for Ser409 p-TDP-43 expression and observed a similar phenotype to that observed with TDP-43 total (Additional file 1: Fig. S3A–C). As previously observed in *Grn*^+*/*+^ and *Grn*^*−/−*^ brains [39], the insoluble fractions of both *Grn*^+*/*+^ and *Grn*^*R493X/R493X*^ exhibited higher levels of full-length p-TDP-43 compared to the soluble fraction (Fig. 4a). Since TDP-43 pathology in this model has previously been demonstrated using TDP-43 immunofluorescent staining [13, 29], we co-stained *Grn*^+*/*+^ and *Grn*^*R493X/R493X*^ brain sections for Tuj1 and TDP-43 to assess neuronal TDP-43 cellular localization (Fig. 4d, e). Tuj1-TDP-43 *Grn*^*R493X/R493X*^ hippocampal/thalamic tilescans showed a major increase in neuronal TDP-43 expression localized to the thalamic VPM/VPL regions compared to *Grn*^+*/*+^. Similar to previous reports, high magnification micrographs of the *Grn*^*R493X/R493X*^ VPM/VPL revealed considerable cytoplasmic TDP-43 accumulation in ventral thalamic neurons (Fig. 4e).

### Neuroinflammation and astrogliosis in the CA3 hippocampus and ventral thalamus of aged *Grn*^*R493X/R493X*^ mice

**Fig. 5:**
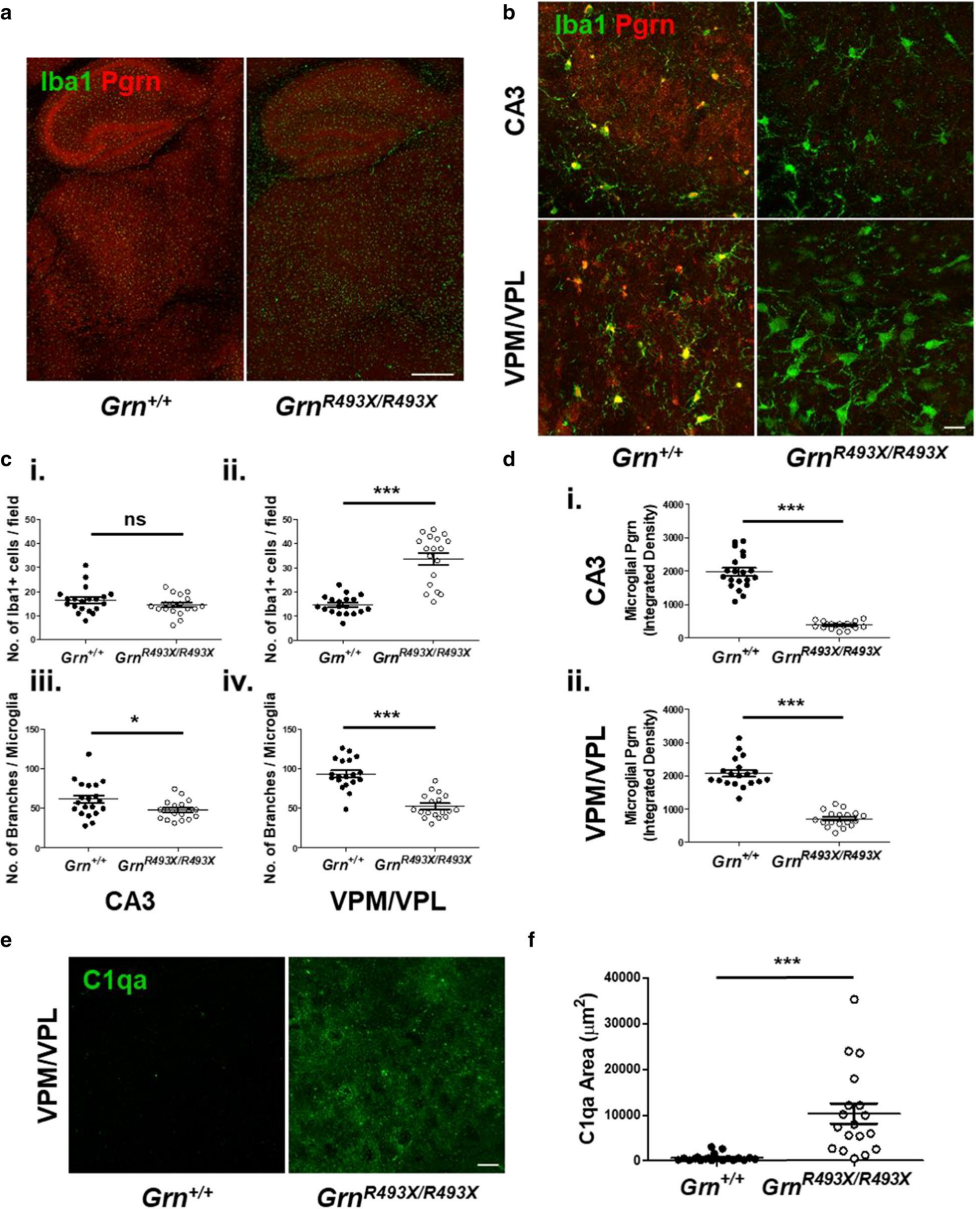
Neuroinflammation in the ventral thalamus of aged *Grn*^*R493X/R493X*^ mice. **a** Representative hippocampal/thalamic tilescans co-stained for Iba1/Pgrn show severe microgliosis in the brain of 18 month old *Grn*^*R493X/R493X*^ mice (scale bar, 500 µm). **b** Representative Iba1/Pgrn co-stained immunofluorescence images of hippocampal CA3 and thalamic VPM/VPL regions in 18 month old *Grn*^+*/*+^ and *Grn*^*R493X/R493X*^ brain sections (scale bar, 20 µm). **c** Quantification of microglial density (i-ii) and branching morphology (iii-iv) in the CA3 and thalamic VPM/VPL regions. **d**, Microglial Pgrn fluorescence intensity in the CA3 (i) and VPM/VPL (ii). **e**–**f**, Representative images and quantification of C1qa staining in the VPM/VPL. *n* = 10 mice were used per sex/genotype (except male *Grn*^*R493X/R493X*^ *n* = 8 and male *Grn*^+*/*+^ Iba1-Pgrn staining of VPM/VPL *n* = 9); values are shown as mean ± SEM; ns not significant, *p < 0.05, ***p < 0.0001 was determined by Student’s *t*-test

Pathologic increases of both microglia and astrocytes in the CA3 hippocampal region and ventral thalamus are well established phenotypes in *Grn*-deficient mice [26, 30]. We sought to characterize neuroinflammation and astrogliosis in aged *Grn*^*R493X/R493X*^ mice. Nguyen et al. previously described a temporal increase in thalamic microglial density in *Grn*^*R493X/R493X*^ mice [29], and this was also observed in our aged *Grn*^*R493X/R493X*^ mice (Fig. 5a). The number of microglia per thalamic VPM/VPL field was significantly greater in mutant mice (Fig. 5b, c.ii); however, no microgliosis was observed in the hippocampal CA3 region (Fig. 5b, c.i). A common feature of pro-inflammatory activated microglia is a morphological transition from a highly ramified state to an amoeboid shape with enlarged soma [9]. To quantify microglia morphology, we conducted skeletal analysis to measure the number of Iba1-positive branches and normalized that to the number of microglia present in a given field to obtain the average branches/microglia (Additional file 1: Fig. S4A, B) [41]. Decreased microglial branching was observed in both the CA3 and VPM/VPL regions of the *Grn*^*R493X/R493X*^ brain, although the phenotype was more pronounced in the VPM/VPL, which exhibited 56.3% ± 6.6% of the branching levels observed in wildtype mice (Fig. 5b, c.iii–iv).

Counterintuitively, increased *GRN* expression has been detected in the frontal and temporal cortices of FTD-*GRN* patients [7]. This observation is believed to result from upregulation of the intact *GRN* allele in hyperactivated, proliferating microglia in degenerating brain regions. In *Grn*^*R493X/R493X*^ mice, upregulation of the mutated alleles could also result in increased basal PTC readthrough which would result in increased full length Pgrn expression. To test this hypothesis, we conducted Pgrn-Iba1 co-staining in *Grn*^+*/*+^ and *Grn*^*R493X/R493X*^ mice to assess whether diseased brain regions in the knockin mice display upregulated microglial Pgrn-R493X expression. We found that microglial Pgrn fluorescent intensity was significantly lower in the CA3 and VPM/VPL of *Grn*^*R493X/R493X*^ mice, suggesting that microglial activation in these regions does not result in substantial basal PTC readthrough or accumulation of truncated Pgrn-R493X (Fig. 5b, d).

**Fig. 6:**
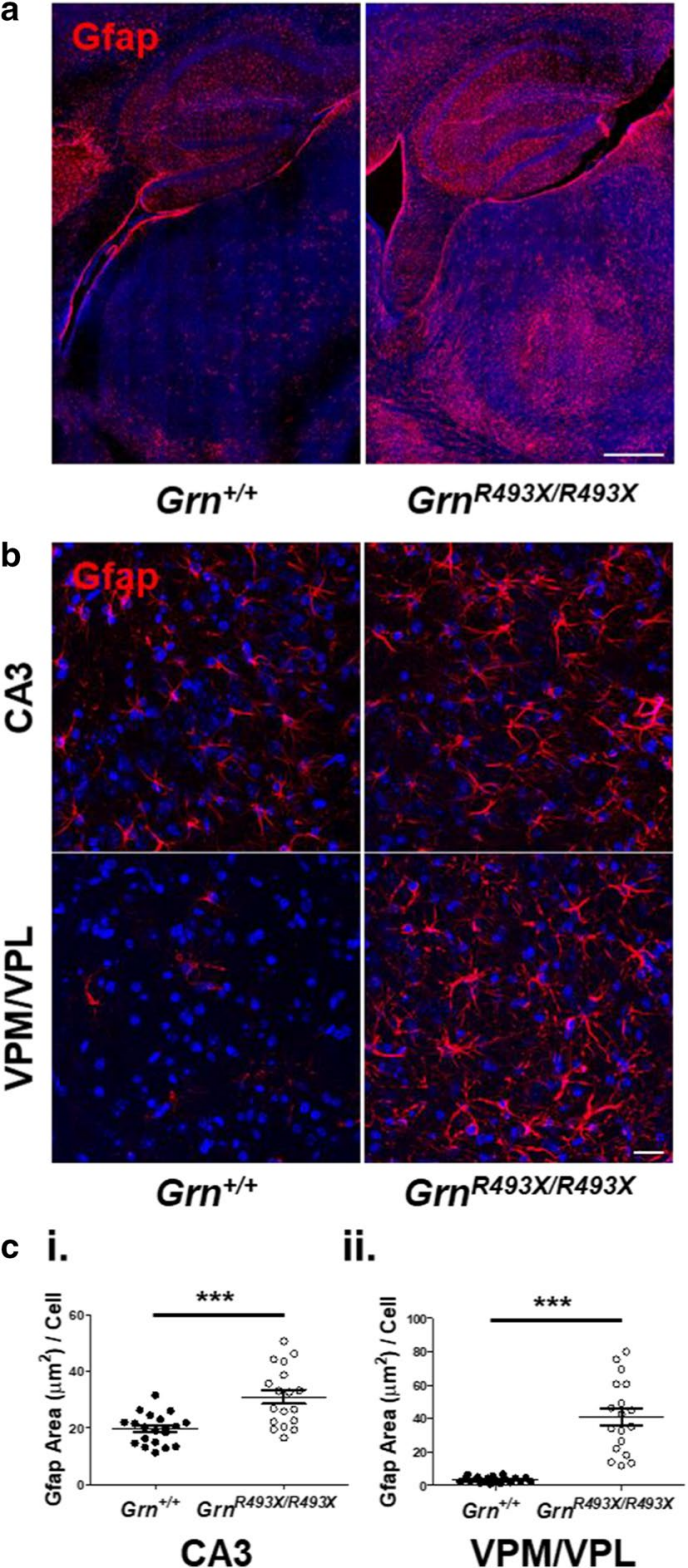
Severe astrogliosis in the ventral thalamus and CA3 hippocampal region of aged *Grn*^*R493X/R493X*^ mice. **a** Representative hippocampal/thalamic tilescans stained for Gfap show severe astrogliosis in 18 month old *Grn*^+*/*+^ and *Grn*^*R493X/R493X*^ brain (scale bar, 500 µm). **b** Representative Gfap immunofluorescence images of hippocampal CA3 and thalamic VPM/VPL regions in 18 month old *Grn*^+*/*+^ and *Grn*^*R493X/R493X*^ brain sections (scale bar, 20 µm). **c** Total Gfap-positive area in CA3 (i) and VPM/VPL (ii) images were quantified and normalized to the total number of cells (DAPI, blue). *n* = 10 mice were used per sex/genotype (except male *Grn*^*R493X/R493X*^ *n* = 8); values are shown as mean ± SEM; ns = not significant, *p < 0.05, ***p < 0.0001, Student’s *t*-test

The innate immune defense system has been previously implicated in the pathology of FTD-*GRN* [26, 42]. Complement-driven synaptic pruning is a critical microglial-mediated neurodevelopmental mechanism [38] that is hyperactivated in the context of *Grn*-deficiency, resulting in selective depletion of thalamic inhibitory synapses in *Grn*^*−/−*^ mice [26]. Complement protein C1qa was significantly increased in the thalamic VPM/VPL of *Grn*^*R493X/R493X*^ mice, similar to that observed in *Grn*^*−/−*^ mice (Fig. 5e, f). Astrogliosis is another hallmark of FTD-*GRN* pathogenesis affecting the same brain regions as microgliosis, as observed by glial fibrillary acidic protein (Gfap) staining in *Grn*^*−/−*^ mice [30, 39]. We present the first characterization of *Grn*^*R493X/R493X*^ astroglial pathology (Fig. 6), observing that Gfap + staining was significantly increased the CA3 and VPM/VPL regions in *Grn*^*R493X/R493X*^ mice (Fig. 6b, c). The astrogliosis phenotype was more striking than microgliosis, spanning both the CA3 and VPM/VPL compared to microgliosis primarily observed in the thalamus.

### Partial preservation of inhibitory synaptic density in the thalamus of aged *Grn*^*R493X/R493X*^ mice

**Fig. 7:**
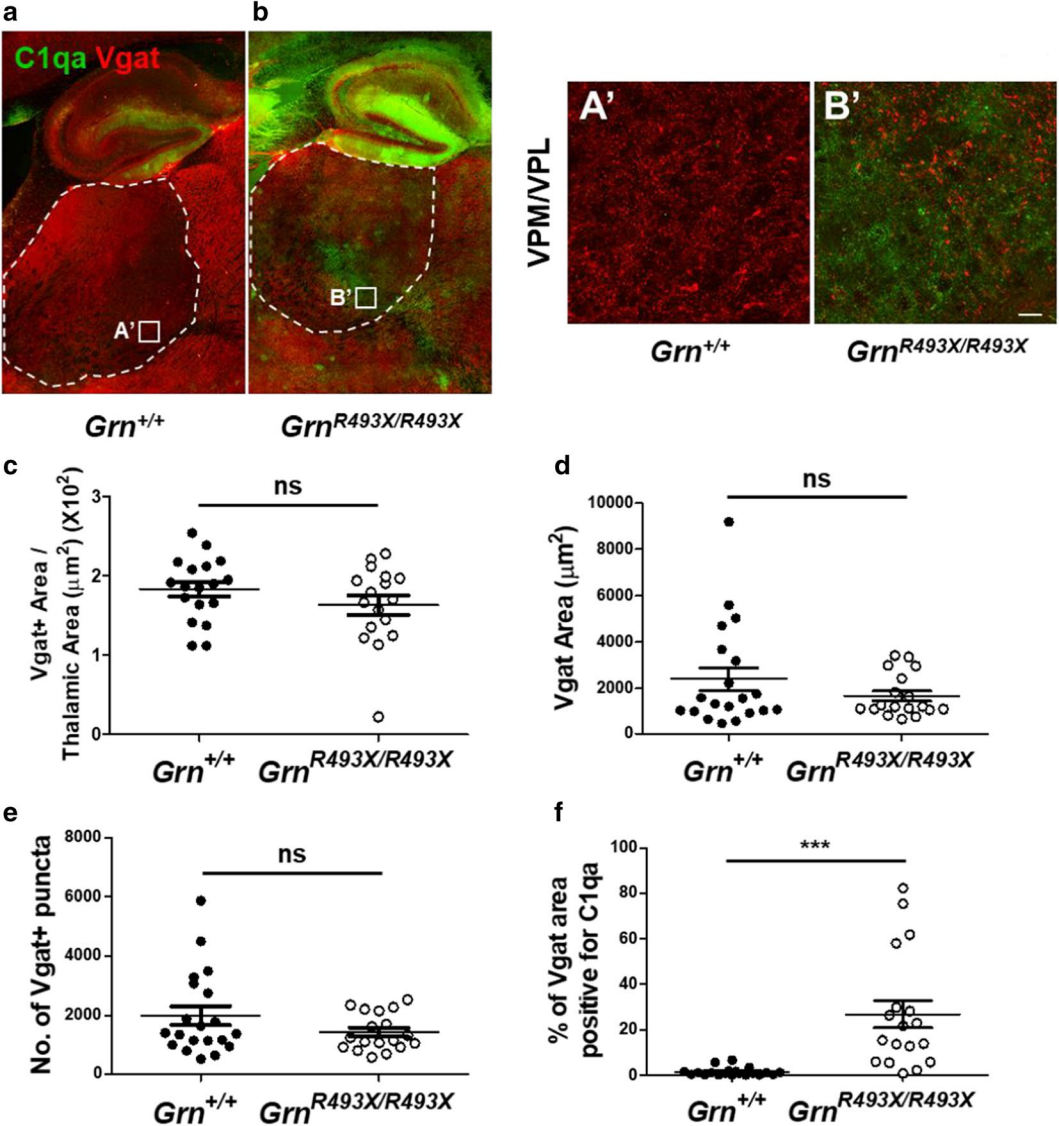
Inhibitory synaptic density is preserved in the thalamus of aged *Grn*^*R493X/R493X*^ mice. **a**, **b** Representative hippocampal/thalamic tilescans co-stained for C1qa and Vgat in 18 month old *Grn*^+*/*+^ and *Grn*^*R493X/R493X*^ mice, the dashed outline depicts area quantified in (**c**) (scale bar, 500 µm). **A**’, **B**’, C1qa-Vgat tilescan inset immunofluorescence images (from **a**, **b**) of thalamic VPM/VPL region in 18 month old *Grn*^+*/*+^ and *Grn*^*R493X/R493X*^ brain sections (scale bar, 20 µm). **c** Quantification of thalamic Vgat-positive synaptic area within hippocampal/thalamic tilescans normalized to thalamic area (white dashed outline). Vgat-positive area (**d**), the number of Vgat-positive synaptic puncta (**e**), and the proportion of Vgat-positive  synaptic area that is positive for C1qa (**f**) was quantified in high-resolution C1qa-Vgat VPM/VPL images (**a**’, **b**’). *n* = 10 mice were used per sex/genotype (except male *Grn*^*R493X/R493X*^ *n* = 8); values are shown as mean ± SEM; ns not significant, ***p < 0.0001, Student’s *t*-test

Given the robust increase in complement C1qa protein deposition observed in the *Grn*^*R493X/R493X*^ ventral thalamus (Fig. 5e, f), we assessed whether this produced a corresponding decrease in inhibitory synaptic density as previously observed in *Grn*^*−/−*^ mice [26]. First, we characterized whole thalamus vesicular gamma-aminobutyric acid transporter (Vgat) synaptic density from hippocampal/thalamic tilescans, and observed a non-significant trend towards lower inhibitory synaptic density in *Grn*^*R493X/R493X*^ mice (Fig. 7a–c). Since the *Grn*^*−/−*^ synaptic phenotype was localized to the ventral thalamus, we further analyzed C1qa-Vgat images of *Grn*^+*/*+^ and *Grn*^*R493X/R493X*^ VPM/VPL regions and found a similar non-significant trend towards lower Vgat synaptic area and number of puncta in *Grn*^*R493X/R493X*^ mice (Fig. 7a’, b’, d–e). Interestingly, this weak trend towards a reduction of thalamic Vgat synaptic density was observed despite 26.9% ± 6.0% of total Vgat-positive synaptic area being co-localized with C1qa in the *Grn*^*R493X/R493X*^ VPM/VPL thalamic regions (Fig. 7f).

### Thalamic neurodegeneration of excitatory neurons in the CNS of aged *Grn*^*R493X/R493X*^ mice

**Fig. 8:**
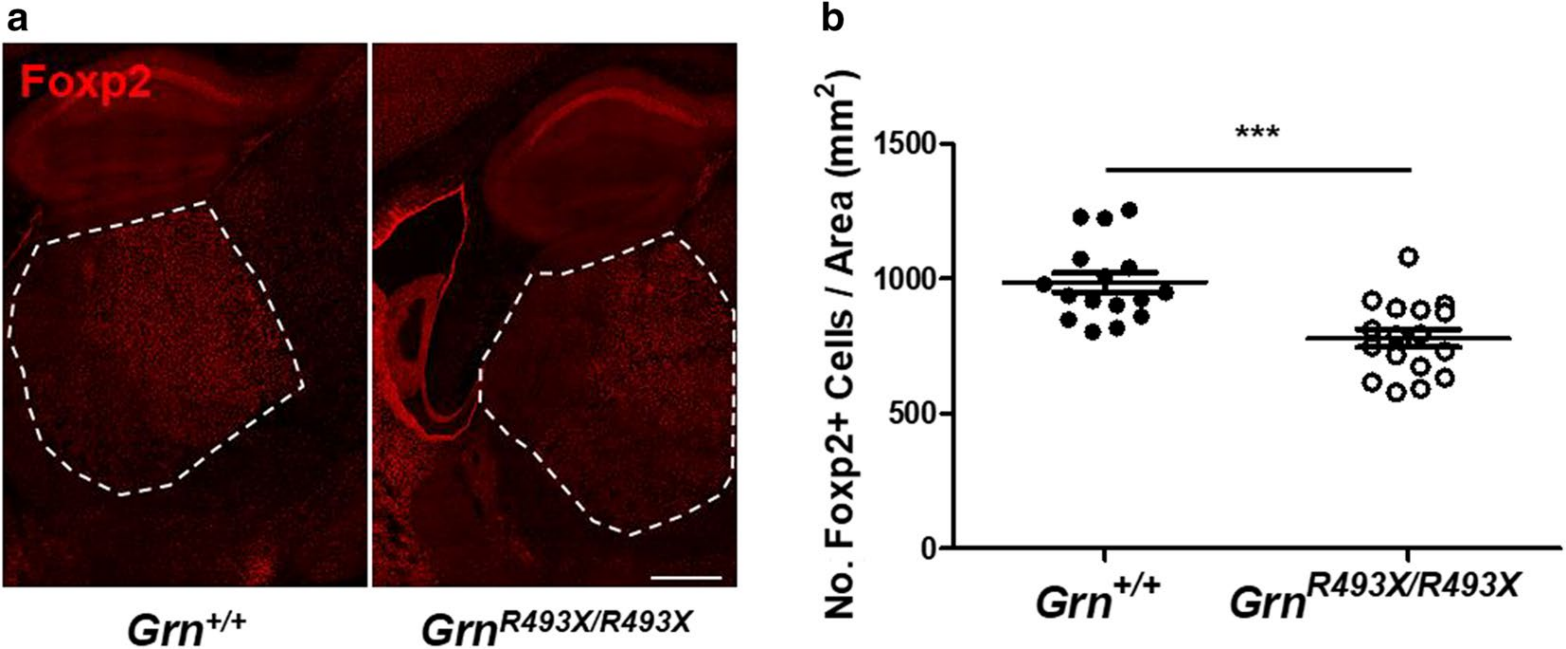
Neurodegeneration of thalamic Foxp2 + excitatory neurons in aged *Grn*^*R493X/R493X*^ mice. **a** Representative hippocampal/thalamic tilescans from 18 month old *Grn*^+*/*+^ and *Grn*^*R493X/R493X*^ mice brain sections stained for Foxp2, the dashed outline depicts area quantified in (**b**) (scale bar, 500 µm). **b** Quantification of total number of thalamic Foxp2-positive nuclei. *n* = 9 male *Grn*^+*/*+^ mice, *n* = 7 female *Grn*^+*/*+^ mice, *n* = 7 male *Grn*^*R493X/R493X*^, and *n* = 10 female *Grn*^*R493X/R493X*^ mice were used; values are shown as mean ± SEM; ***p < 0.0001, Student’s *t*-test

A recent single-nuclei RNA sequencing (snRNA-seq) study by Zhang et al. characterizing disease progression in *Grn*^*−/−*^ mice from 2 to 19 months of age, discovered a selective decrease in the abundance of excitatory neuron markers from 12 to 19 months of age [42]. We attempted to replicate these findings in *Grn*^*R493X/R493X*^ mice by performing Foxp2 immunofluorescence staining and quantifying the number of Foxp2-positive thalamic neurons. 18 month old *Grn*^*R493X/R493X*^ thalami exhibited a significant reduction in the number of Foxp2-positive excitatory neurons compared to *Grn*^+*/*+^ mice (21.0% ± 4.3%), confirming select neurodegeneration in this model (Fig. 8a, b).

### Aged *Grn*^*R493X/R493X*^ mice exhibit an increased anxiety phenotype

**Fig. 9:**
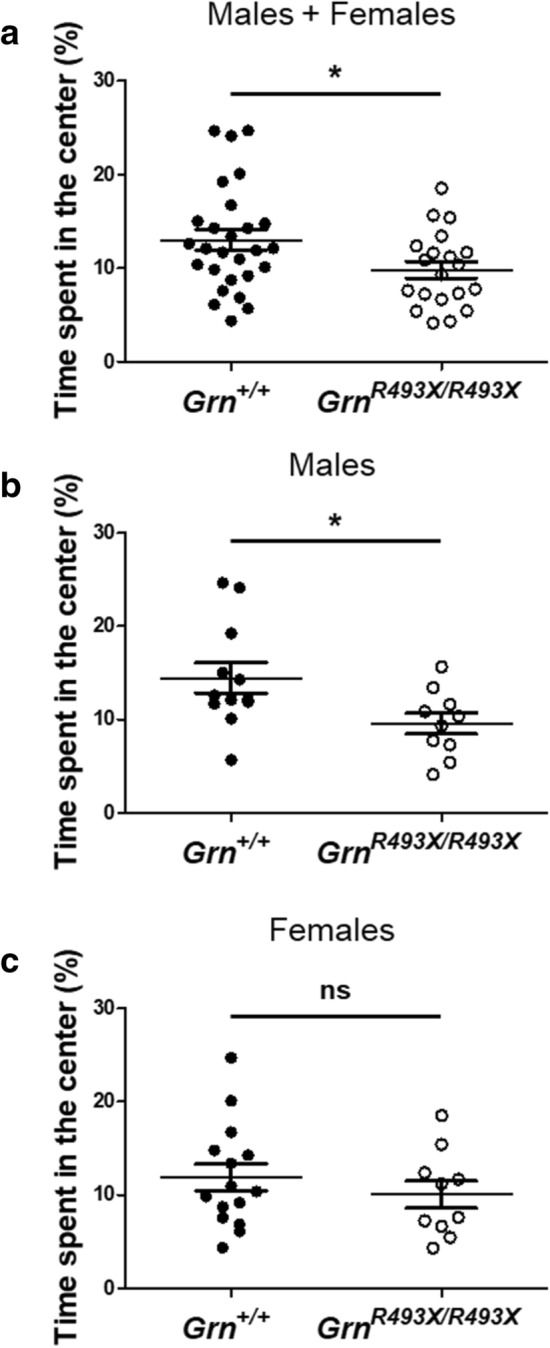
Aged male *Grn*^*R493X/R493X*^ mice exhibit an increased anxiety phenotype. Proportion of time male/female (**a**), male (**b**), and female (**c**) *Grn*^+*/*+^ and *Grn*^*R493X/R493X*^ mice spent inside the center region of an open-field arena over a 10 min trial. Male *Grn*^+*/*+^ *n* = 12, female *Grn*^+*/*+^ *n* = 15, male *Grn*^*R493X/R493X*^ *n* = 10, and female *Grn*^*R493X/R493X*^ *n* = 10; values are shown as mean ± SEM; ns = not significant, *p < 0.05, Student’s *t*-test

Several behavioral phenotypes have been identified in *Grn*^+/−^ and *Grn*^*−/−*^ mice, including deficits in social dominance, excessive grooming, and increased anxiety [2, 23, 26, 29, 30]. The initial *Grn*^*R493X/R493X*^ characterization found nearly identical onset and progression of obsessive-compulsive-like grooming behavior in *Grn*^*−/−*^ and *Grn*^*R493X/R493X*^ mice resulting in severe skin lesions, which likely contributed to their 30% lower median survival rate [29]. Though we did not specifically aim to quantify these phenotypes in our mice, we did observe a trend towards more animal facility health updates reporting lesions and grade 4 whisker barbering in *Grn*^*R493X/R493X*^ mice (colonies: *Grn*^+*/*+^ 5/199 mean age: 66.2 ± 1.8 weeks vs. *Grn*^*R493X/R493X*^ 16/233 mean age: 49.4 ± 1.8 weeks) often requiring euthanasia likely attributable to excessive grooming behavior. The open-field test has been used to establish the increased male-specific anxiety phenotype in *Grn*^*−/−*^ mice by quantifying the time mice spent in the central vs peripheral regions of the open-field [23, 30]. *Grn*^*R493X/R493X*^ mice spent significantly less time in the central region of the open-field compared to *Grn*^+*/*+^ mice (Fig. 9a). We conducted a sex-specific analysis to further evaluate whether this anxiety phenotype was limited to male *Grn*^*R493X/R493X*^ mice and found that male knockin mice spent significantly less time in the central zone than *Grn*^+*/*+^ males, while female *Grn*^+*/*+^/*Grn*^*R493X/R493X*^ mice spent similar amounts of time in each region (Fig. 9b, c). To probe whether any of the previously presented neuropathological phenotypes show any sex-dependent effects, we conducted further sex-specific analyses of these data and failed to observe any statistically significant differences between sexes (Additional file 1: Figs. S5, S6, S7).

## Discussion

We provide the first detailed analysis of lysosomal dysfunction and selective loss of thalamic excitatory neurons in the brains of aged *Grn*^*R493X/R493X*^ mice. Since disrupted lysosomal homeostasis is a pathological hallmark of *Grn*^*−/−*^ mice, we chose to evaluate several previously established brain lysosomal phenotypes in our aged *Grn*^*R493X/R493X*^ cohort. The CA3 hippocampal and thalamic VPM/VPL brain regions of aged *Grn*^*R493X/R493X*^ mice displayed striking expansions of their lysosomal compartments. We further evaluated aged knockin mice for global changes in brain lysosomal function and impairments in autophagy, identifying overexpression of both the pro- and mature-forms of lysosomal protease Ctsd and impaired clearance of autophagolysosomes as indicated by an increased LC3-II:LC3-I ratio, both of which have been previously observed in aged *Grn*^*−/−*^ mice [6, 16]. Notably, evidence of lysosomal dysfunction has been demonstrated in *Grn*^*−/−*^ mice as young as 2 months of age [24]. Future efforts may seek to understand whether the presence of a semi-functional, truncated Pgrn-R493X might delay the onset of this early lysosomal phenotype.

*GRN* deficiency is associated with nuclear to cytoplasmic translocation of TDP-43, ultimately resulting in the formation of insoluble neuronal inclusions [1, 10]. The redistribution of TDP-43 from the nucleus to the cytoplasm has also been observed in *Grn*^*−/−*^ mice, a process which may involve excessive neuroinflammation and dysfunctional autophagolysosomal and ubiquitin–proteasome systems [13, 29, 39, 42]. Similarly to these findings, we found TDP-43 pathology limited to ventral thalamic neurons in *Grn*^*R493X/R493X*^ mice, which exhibited intense nuclear to cytoplasmic TDP-43 translocation. We further observed that *Grn*^*R493X/R493X*^ soluble hemi-brain lysates contained decreased TDP-43, potentially indicating that a proportion of the soluble TDP-43 pool had transitioned into an insoluble form. This phenomenon was observed in a mutant TDP-43 mouse model where soluble TDP-43 increased while insoluble TDP-43 decreased upon overexpression of PGRN [5]. However, we failed to observe a corresponding increase in the insoluble levels of TDP-43 and were unable to detect p-TDP-43 in either soluble or insoluble lysates, both of which have been previously found in aged *Grn*^*−/−*^ mice [17, 39]. The former most likely relates to our finding that TDP-43 pathology is largely limited to ventral thalamic neurons, and the phenotype may thus be lost through dilution when assessing whole-brain lysate. Other discrepancies are less clear and may relate to inherent differences in mouse models as well as the particular age chosen for analysis.

*Grn*^*R493X/R493X*^ mice exhibit age-dependent microgliosis that begins around 6 months of age, reaching a peak at 12 months, and is maintained until 18 months of age. We observed significant neuroinflammation in the ventral thalamus of *Grn*^*R493X/R493X*^ mice, including astro- and microglial expansion and morphological transition into a proinflammatory state. This observation is important because we show that the chronically inflamed ventral thalamic brain region also develops TDP-43 proteinopathy, providing additional support to the growing evidence demonstrating that factors secreted by *Grn* deficient microglia directly induce cytoplasmic accumulation of TDP-43 [42]. These inflammatory mediators include the innate immune system complement system (C1qa, C3, etc.) which have been implicated in *Grn*-deficient microglial mediated neurotoxicity. Although most of the literature has focused on examining microglial-driven neuropathological mechanisms, a recent study by Guttikonda et al. conducted using human induced pluripotent stem cell-derived neuron-astrocyte-microglia tricultures suggested that reciprocal C3 signalling between microglia and astrocytes is critical to driving excessive microglial C1qa complement protein expression and secretion [19]. Therefore, future studies assessing the role of *Grn*-deficient astrocytes in driving disease pathophysiology may be critical to identifying novel therapeutic strategies.

Microglial-mediated activation of the complement pathway has been directly and indirectly implicated in driving thalamic neurodegeneration in *Grn*^*−/−*^ mice through selective targeting of both inhibitory synapses and excitatory neurons for elimination [26, 42]. Despite observing robust C1qa tagging of Vgat + synapses as previously seen in *Grn*^*−/−*^ mice, neither *Grn*^*R493X/R493X*^ whole thalami or the VPM/VPL region showed a significant decrease in inhibitory synaptic density. However, there was a trend towards lower levels of Vgat-positive synapses in these regions. It is possible that low basal Pgrn-R493X expression in *Grn*^*R493X/R493X*^ microglia limited their voracity for inhibitory synapses, but this is not known. A recent snRNA-seq study found that selective loss of excitatory neurons in the ventral thalamus of *Grn*^*−/−*^ mice could be rescued by simultaneous deletion of both *C1qa* and *C3* complement genes [42]. The authors proposed that elevated C1qa and C3 in this brain region result in increased membrane attack complex formation on neuronal surfaces, which permeabilizes their plasma membranes, triggering apoptosis [42]. Similarly, our aged *Grn*^*R493X/R493X*^ displayed loss of thalamic Foxp2-positive excitatory neurons, perhaps suggesting that a loss of excitatory neurons precedes the synaptic pruning phenotype in this model. Nevertheless, it remains unclear how these complement-mediated neurodegenerative mechanisms selectively target inhibitory synapses and excitatory neurons.

*Grn*-deficient mice develop behavioral abnormalities impacting social interactions, grooming frequency, and anxiety levels [2, 23, 26, 29, 30]. *Grn*^*R493X/R493X*^ mice were no different, exhibiting a male-specific increased anxiety phenotype previously reported in *Grn*^*−/−*^ mice [23, 30]. Study of neuropathological correlates of behavioral disturbances in *Grn*^*−/−*^ have connected decreased inhibition of the thalamocortical circuit to their obsessive-compulsive-like grooming phenotype [26]. Since we observed significant thalamic pathology in aged *Grn*^*R493X/R493X*^ mice, we analyzed the major neuropathological phenotypes presented here for sexual dimorphism to assess whether increased pathology might explain the male-predominant anxiety phenotype. While we found that levels of inhibitory synaptic density in the thalamic VPM/VPL regions exhibited a strong trend towards a sex-dependent phenotype in both *Grn*^+/+^ and *Grn*^*R493X/R493X*^ mice (Additional file 1: Fig. S7C), with males displaying elevated inhibitory synaptic density compared to females, we did not observe sexually dimorphic FTD-related pathology in *Grn*^*R493X/R493X*^ mice. Similar efforts have been made to identify the neurological basis for the increased susceptibility of male *Grn*-deficient mice to the development of an increased anxiety phenotype [8, 32]. These studies found that *Grn* expression is upregulated in the ventromedial hypothalamic nucleus in response to androgen and estrogen sex-hormones and that Pgrn is an essential mediator of male sexual differentiation in the developing brain. Since *Grn*^+*/*+^ female mice generally exhibit elevated anxiety compared to *Grn*^+*/*+^ males, it has been proposed that a lack of *Grn* expression during sexual differentiation results in at least partial fulfillment of the default female neurodevelopmental program [23]. Evidence supporting this hypothesis includes the observation that *Grn*^*−/−*^ mice lack the sexually dimorphic trait of differential locus ceruleus (LC) volume, which is normally larger in *Grn*^+*/*+^ females [18]. Because the LC is an important regulator of stress-induced anxiety responses, it is possible that a relatively enlarged LC in male *Grn*^*−/−*^ compared to male *Grn*^+/+^ mice could predispose them to increased anxiety [28]. These studies suggest profound developmental changes in the brain as a result of *Grn* deficiency. Improved preclinical methodology, including the use of hiPSCs, may allow future studies to probe these mechanisms which could reveal processes far upstream of known FTD pathology that may prove central to FTD-*GRN* pathophysiology.   

Taken together, a striking finding in both aged *Grn*^*R493X/R493X*^ mice and other *Grn*^*−/−*^ models is the pronounced involvement of select thalamic regions. For the clinician this might be curious as neurodegeneration in the frontal, temporal, and parietal lobes is widely recognized as driving clinical symptoms across FTLD syndromes. However, a recent study in preclinical *GRN* carriers found prominent hyperconnectivity between the thalamus and cortical hub regions in several intrinsic connectivity networks, assessed by functional magnetic resonance imaging [25]. The implications of these findings are not fully clear, but abnormal thalamic physiology, which is robustly demonstrated in FTD-*GRN* mouse models either through histology or electrophysiology, may have important implications for the earliest changes in human FTD-*GRN*. As such, interventions to reverse these pathologic changes in mice may have important translational value.

## Conclusions

In conclusion, our aged cohort of *Grn*^*R493X/R493X*^ mice displayed several pathologic phenotypes, including lysosomal dysfunction and select thalamic synaptic degeneration not previously described in this model, but in line with observations in other *Grn*^*−/−*^ model mice. Our characterization of aged *Grn*^*R493X/R493X*^ mice provides the field with further insight into neuropathological phenotypes that may be used to better define the mechanisms underlying FTD-*GRN*, and evaluate the preclinical efficacy of novel therapeutics to target relevant nonsense mutations leading to FTD-*GRN*.

## Declarations

## Supplementary Information

**Additional file 1.** Supplementary Figures and Extended Methods.

## Data Availability

The datasets used and/or analysed during the current study are available from the corresponding author upon request.

## Abbreviations

FTLD: Frontotemporal lobar degeneration
FTD: Frontotemporal dementia
GRN: Human progranulin gene
Grn: Murine progranulin gene
LOF: Loss of function
NCL: Neuronal ceroid lipofuscinosis
PGRN: Human progranulin
UBC: University of British Columbia
PCR: Polymerase chain reaction
PBS: Phosphate-buffered saline
RIPA: Radioimmunoprecipitation assay
ELISA: Enzyme-linked immunofluorescent assay
D-PBS: Dulbecco’s phosphate-buffered saline
DAPI: 4′,6′-Diamidino-2-phenylindole
SDS-PAGE: Sodium dodecyl sulphate–polyacrylamide gel electrophoresis
TBS: Tris-buffered saline
TBS-T: Tris-buffered saline with 0.1% Tween-20
Pgrn: Murine progranulin
PTC: Premature termination codon
CNS: Central nervous system
RT: Room temperature
Ctsd: Cathepsin D
TDP-43: TAR DNA-binding protein 43
CTF: C-terminal fragment
Gfap: Glial fibrillary acidic protein
snRNA-seq: Single-nuclei RNA sequencing
