# Supplementary material for: Neuropathological and behavioral characterization of aged Grn R493X progranulin-deficient frontotemporal dementia knockin mice
Source: Acta Neuropathol Commun. 2021 Apr 1;9:57. doi: 10.1186/s40478-021-01158-x (PMC8017751; doi:10.1186/s40478-021-01158-x)
Supplement: Supplementary file 1 — Additional file 1. Supplementary Figures and Extended Methods. [file 40478_2021_1158_MOESM1_ESM.pdf]

Supplemental Figures

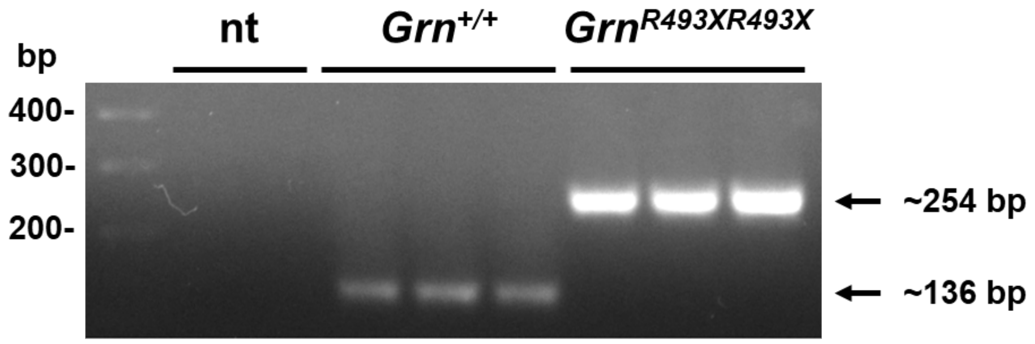

Fig. S1. PCR genotyping results confirm the presence of the homozygous *Grn*^{R493X} knock-in alleles in *Grn*$^{R493X/R493X}$ mice. nt = no template, bp = base pair.

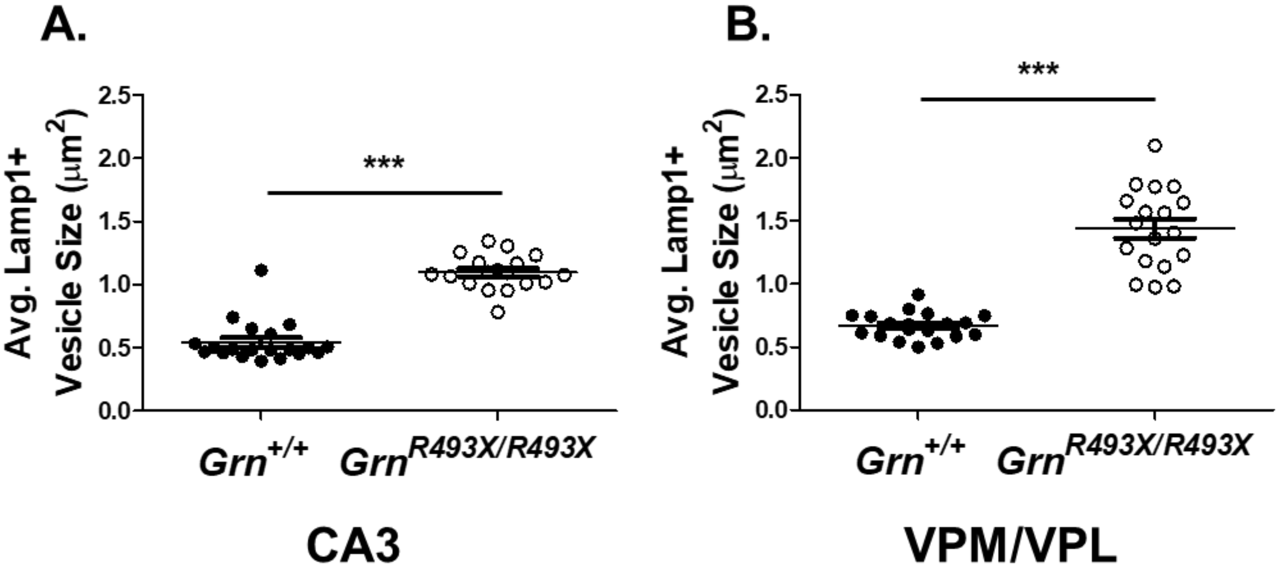

Fig. S2. Enlarged lysosomes in the ventral thalamus and CA3 hippocampal region of aged *Grn*$^{R493X/R493X}$ mice. Average Lamp1+ vesicle size was quantified in images of *Grn*$^{+/+}$ and *Grn*$^{R493X/R493X}$ CA3 (A) and VPM/VPL (B). For immunofluorescence staining n=10 mice were used per sex/genotype (except male *Grn*$^{R493X/R493X}$ n=8); values are shown as mean ± SEM; *** $p < 0.0001$ was determined by Student's *t*-test.

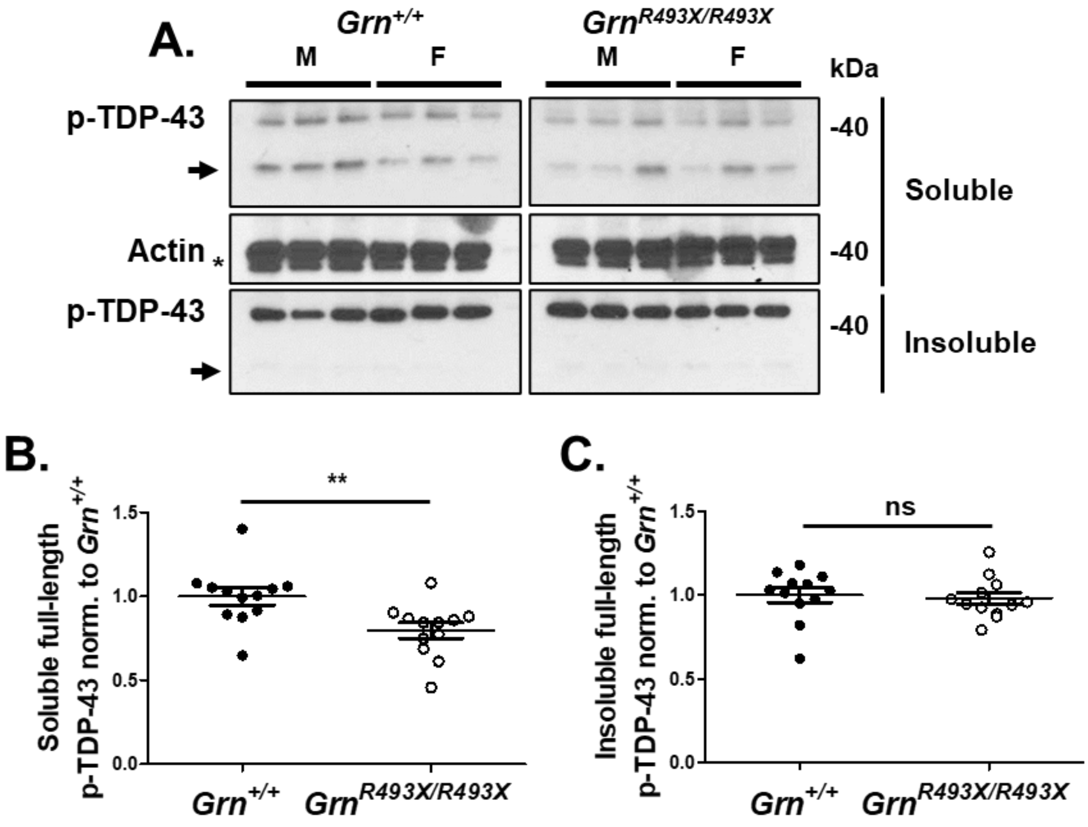

Fig. S3. Absence of global p-TDP-43 proteinopathy phenotype in aged *Grn*$^{R493X/R493X}$ CNS. Representative western blots of hemibrain RIPA-soluble and -insoluble lysates from 18 month old *Grn*$^{+/+}$ and *Grn*$^{R493X/R493X}$ mice probed for TDP-43 expression. A, Expression of full-length p-TDP-43 in *Grn*$^{+/+}$ and *Grn*$^{R493X/R493X}$ hemibrains in soluble and insoluble lysates was analyzed by western blotting, using RIPA-soluble actin as the loading control (no actin detected in insoluble UREA fraction). Arrows indicate TDP-43 CTFs, and the * demarks a remnant TDP-43 signal observed upon reprobing stripped RIPA-soluble TDP-43 blot with actin antibody. Densitometric quantification of CNS-wide full-length p-TDP-43 expression in soluble (B) and insoluble (C) lysate fractions were normalized to RIPA-soluble actin and *Grn*$^{+/+}$ levels. n=6 mice were used per sex/genotype; values are shown as mean ± SEM; ns = not significant, ** $p < 0.01$, was determined by Student's *t*-test.

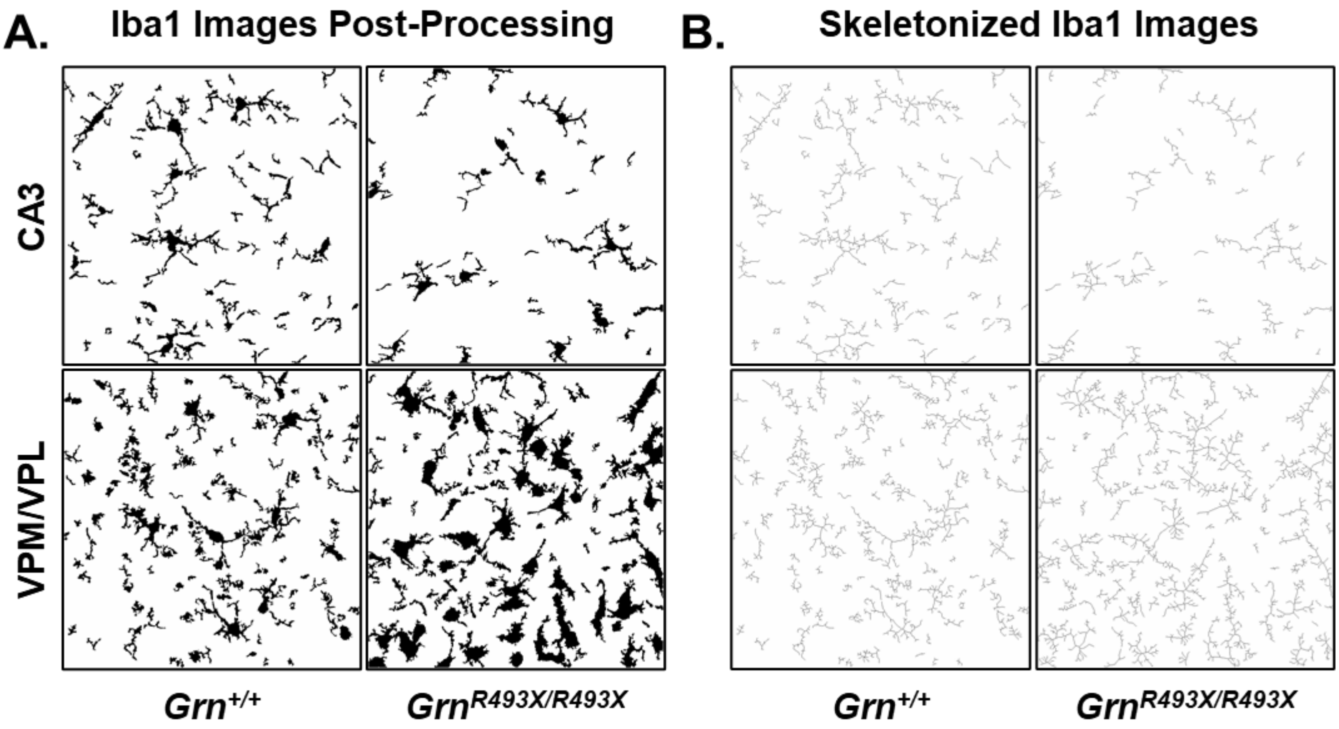

Fig. S4. Microglial skeletal analysis method. Iba1 immunofluorescent images were processed in Fiji as described in supplemental methods section (A) and skeletonized using the Fiji Analyze Skeleton (2D/3D) plugin (B).

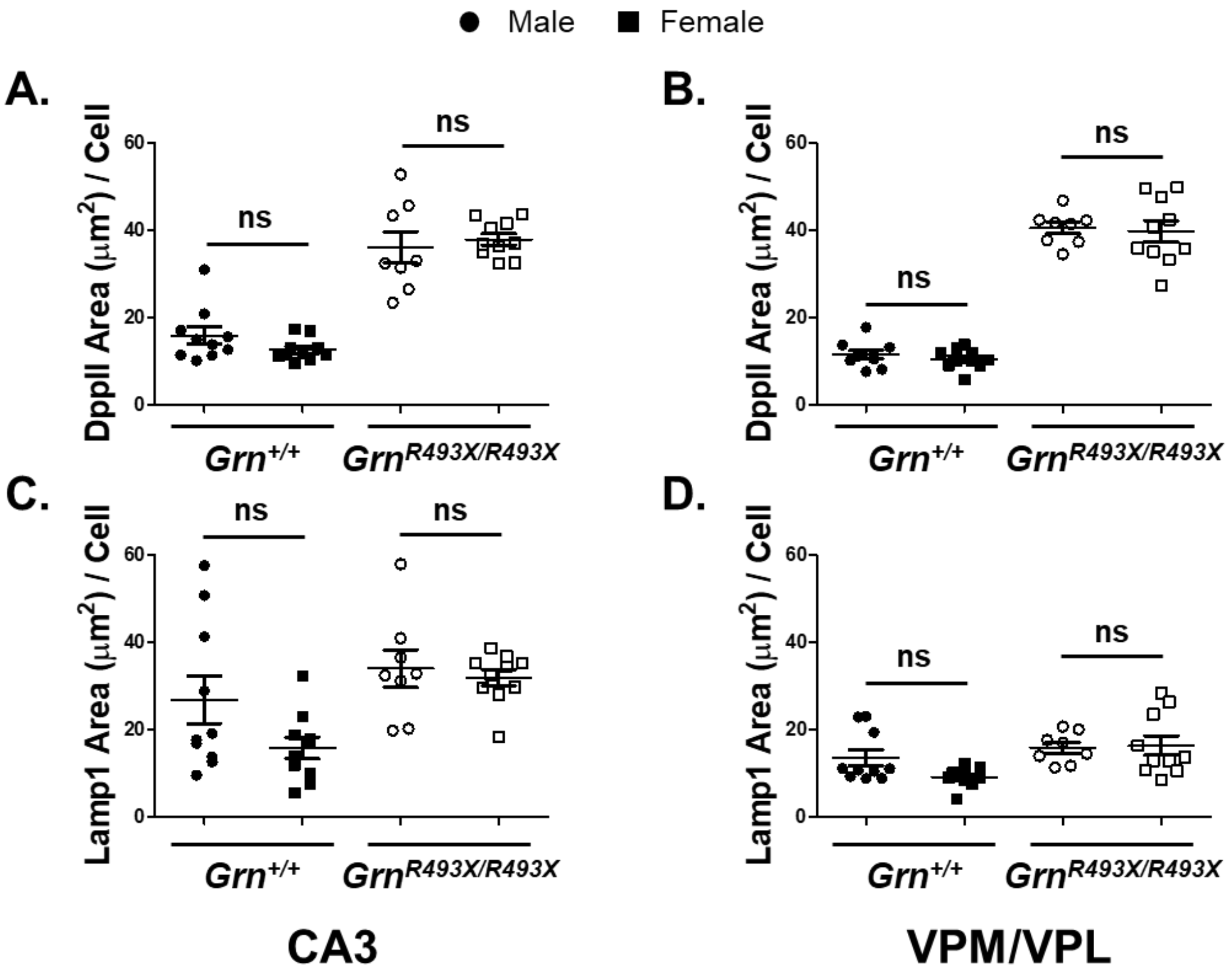

Fig. S5. ***$Grn^{R493X/R493X}$* CNS lysosomal dysfunction does not exhibit a sex-specific phenotype.** DppII+ (A-B) and Lamp1+ (C-D) area/cell in both male and female *$Grn^{+/+}$* and *$Grn^{R493X/R493X}$* CA3 and VPM/VPL images were quantified and normalized to the total number of cells (DAPI). For immunofluorescence staining n=10 mice were used per sex/genotype (except male *$Grn^{R493X/R493X}$* n=8); values are shown as mean ± SEM, ns = not significant was determined by one-way ANOVA with Tukey's multiple comparison test.

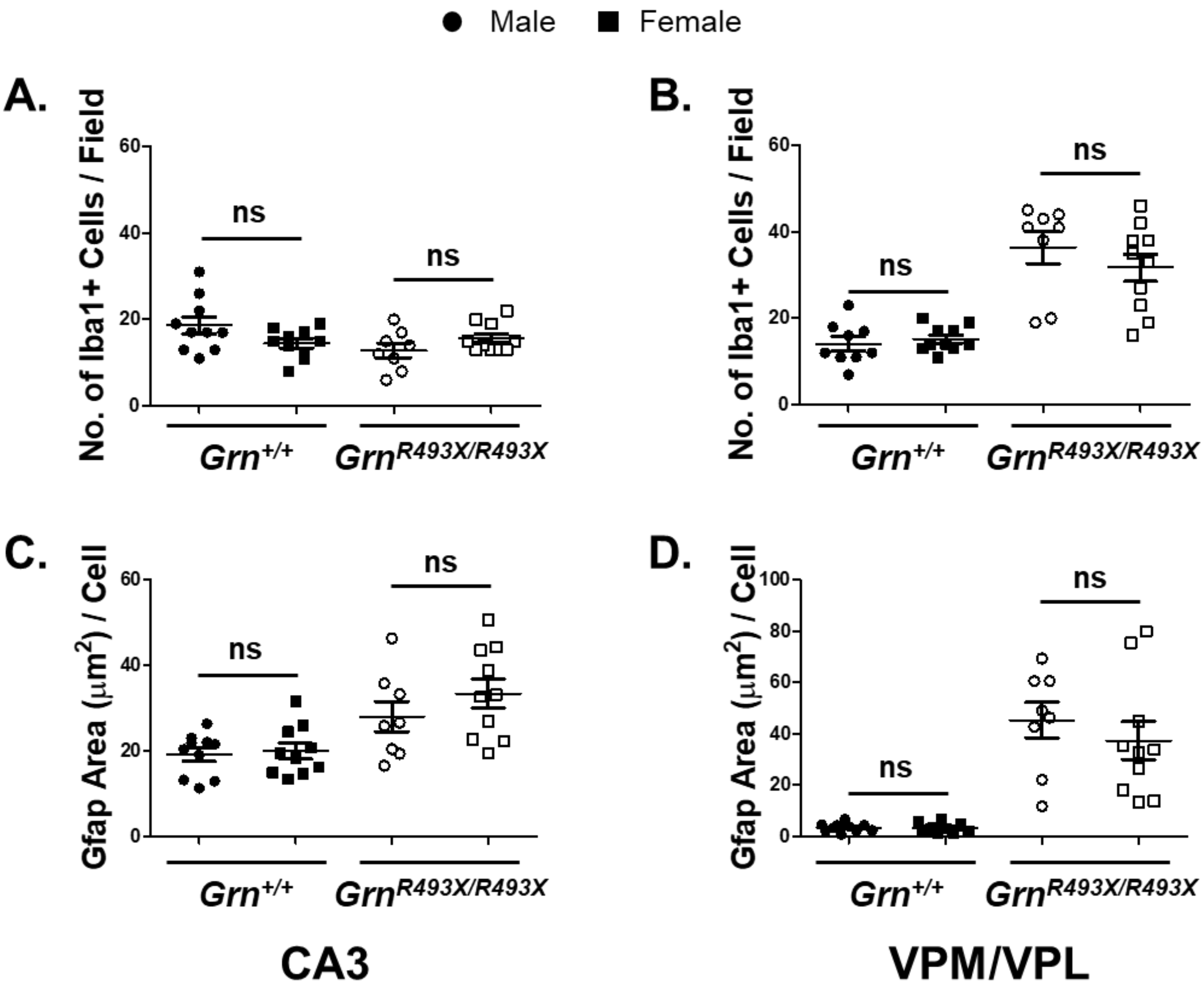

Fig. S6. *Grn*$^{R493X/R493X}$ microgliosis and astrogliosis does not exhibit a sex-specific phenotype. Iba1+ microglial density was quantified in both male and female *Grn*$^{+/+}$ and *Grn*$^{R493X/R493X}$ CA3 (A) and VPM/VPL (B). Gfap+ astroglial density was quantified in both male and female *Grn*$^{+/+}$ and *Grn*$^{R493X/R493X}$ CA3 (C) and VPM/VPL (D). n=10 mice were used per sex/genotype (except male *Grn*$^{R493X/R493X}$ n=8 male and *Grn*$^{+/+}$ Iba1 staining of VPM/VPL n=9); values are shown as mean ± SEM, ns = not significant was determined by one-way ANOVA with Tukey's multiple comparison test.

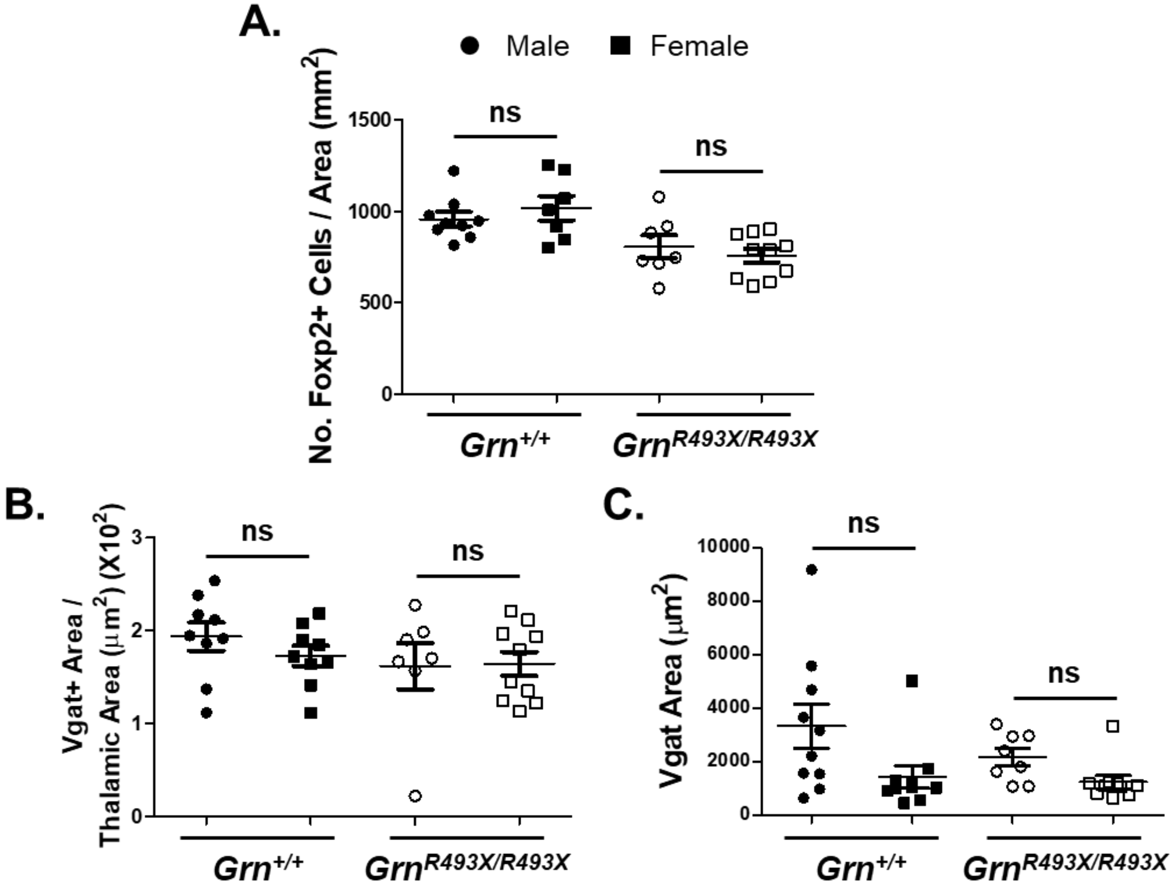

Fig. S7. *Grn*$^{R493X/R493X}$ neurodegeneration of thalamic excitatory neurons and inhibitory synaptic pruning does not exhibit a sex-specific phenotype. A, Quantification of total number of thalamic Foxp2+ nuclei in male and female *Grn*$^{+/+}$ and *Grn*$^{R493X/R493X}$. Quantification of total thalamic (B) and VPM/VPL (C) Vgat+ inhibitory synaptic area in male and female *Grn*$^{+/+}$ and *Grn*$^{R493X/R493X}$. Total thalamic Foxp2 (A) n=9 male *Grn*$^{+/+}$ mice, n=7 female *Grn*$^{+/+}$ mice, n=7 male *Grn*$^{R493X/R493X}$, and n=10 female *Grn*$^{R493X/R493X}$ mice, total thalamic Vgat (B) n=9 male *Grn*$^{+/+}$ mice, n=9 female *Grn*$^{+/+}$ mice, n=7 male *Grn*$^{R493X/R493X}$, and n=10 female *Grn*$^{R493X/R493X}$ mice, and VPM/VPL Vgat (C) n=10 mice were used per sex/genotype (except male *Grn*$^{R493X/R493X}$ n=8 male); values are shown as mean ± SEM, ns = not significant was determined by one-way ANOVA with Tukey’s multiple comparison test.

Supplemental Methods

Histological quantification

The total area (μm^2) of lysosomal marker (Lamp1 and DppII) staining per CA3 or VPM/VPL field was quantified using Fiji by applying a uniform threshold to all images and watershed segmentation to distinguish individual lysosomal vesicles. The analyze particle function was used to quantify areas ranging from 0.05-infinity μm^2 providing both total Lamp1+/DppII+ area and average lysosomal vesicle size. Total Pgrn staining area was quantified by applying a uniform threshold across all images and analyzing particle areas 0-infinity μm^2. Microgliosis was quantified by assessing both total Iba1+ area and the number of Iba1+ cells; this was determined by applying a uniform threshold and analyzing particles with an area of 0.25-infinity μm^2 and 20-infinity μm^2, respectively. Astrogliosis was similarly quantified by measuring total Gfap+ area in uniformly thresholded images using the analyze particles tool (0.25-infinity μm^2). Thalamic VPM/VPL total C1qa complement staining area was quantified by applying a uniform threshold across all images and analyzing particle areas 0-infinity μm^2. Ventral thalamic density of Vgat+ synapses was determined by quantifying both total Vgat+ area and the number of Vgat+ puncta. To quantify these metrics, Vgat images were uniformly thresholded and segmented using the watershed function and particles limited to a 0.3-5 μm^2 area were analyzed. The % of Vgat+ synaptic area co-stained for C1qa was determined by generating regions of interest (ROIs) of Vgat+ synapses using the above image processing and analyze particle specifications. These Vgat ROIs were then applied to corresponding co-stained C1qa images and any C1qa+ staining outside of ROIs was cleared using the clear outside function. The remaining intra-ROI C1qa staining was uniformly thresholded and quantified by analyzing particle areas 0-infinity μm^2. The total C1qa area within Vgat ROIs was divided by total Vgat area and X100 to obtain % of Vgat+ synaptic area co-stained for C1qa. For data normalized on

a per cell basis, the number of DAPI+ nuclei were quantified per field by applying a uniform threshold to all images and watershed segmentation to distinguish individual nuclei. The analyze particle function was used to quantify areas ranging from 25-infinity μm^2 to determine the total number of cells per field.

Foxp2+ excitatory neuron and Vgat+ synaptic total thalamic density was obtained from MIPs generated from z-stack tilescans containing both the hippocampal and thalamic brain structures (40X magnification, 0.6 zoom, 8 x 12 tilescan). To obtain these results the polygon selection tool was used to draw a boundary surrounding the thalamus to measure the total thalamic area (mm^2). Any Foxp2+ or Vgat+ staining outside of the thalamic boundary was eliminated using the clear outside function and a uniform threshold was applied to each tilescan image. Following watershed segmentation to allow for clear detection of individual Foxp2+ nuclei and Vgat+ synaptic puncta, the number of particles 50-200 μm^2 (Foxp2) or 7.75-25 μm^2 (Vgat) in size were analyzed and normalized to total thalamic area.

Microglial skeletal analysis was conducted using the Fiji Analyze Skeleton (2D/3D) plugin using a modified version of a previously described protocol (1). Pre-analysis Iba1 image processing required adjusting image brightness maximum (-40 to 215), applying unsharp mask (radius 3 and mask weight 0.6), and reducing background noise using the despeckle tool. These processed images were then uniformly thresholded, further despeckled, processed using the binary Close- tool, and background noise was reduced further using the remove outliers function. The analyze particles function was then used to generate ROIs masking Iba1+ staining of a 10-infinity μm^2 area and add them to the ROI manager. The original image was then reopened and the initial Iba1 image processing steps were reapplied (brightness, unsharp mask, and despeckled). All ROIs were then selected in the ROI manager and applied to this

image using the OR (combine) function. Any Iba1+ staining outside of the applied ROIs was eliminated using the clear outside function and a threshold of 1-255 was applied to completely fill in the ROIs generating a clean binary image of microglial cell shapes. This image was then skeletonized using the binary processing tool and analyzed with the Analyze Skeleton (2D/3D) plugin (prune cycle: none, checked boxes = ‘show detailed info’ and ‘display labelled skeletons’) to determine the total number of microglial branches per field. To quantify microglial Pgrn fluorescent intensity (integrated density) from Iba1-Pgrn co-staining microglial ROIs were generated from Iba1 images according to the above skeletal analysis method. These ROIs were applied to their corresponding Pgrn co-stained image and any Pgrn+ staining outside of the applied microglial ROIs was removed using the clear outside function. Pgrn integrated density in each microglial ROI was then measured from the ROI manager. The calculated integrated densities for all microglial ROIs per image were averaged providing the Pgrn microglial fluorescent intensity per field.

References

(1) Young K, Morrison H. Quantifying microglia morphology from photomicrographs of immunohistochemistry prepared tissue using ImageJ. JoVE (Journal of Visualized Experiments) 2018(136):e57648.
